# ProC6C, a novel multi-stage malaria vaccine, elicits functional antibodies against the minor and central repeats of the Circumsporozoite Protein in human adults

**DOI:** 10.3389/fimmu.2024.1481829

**Published:** 2024-11-01

**Authors:** Jordan Plieskatt, Ebenezer Addo Ofori, Mohammad Naghizadeh, Kazutoyo Miura, Yevel Flores-Garcia, Nis Borbye-Lorenzen, Alfred B. Tiono, Kristin Skogstrand, Issaka Sagara, Fidel Zavala, Michael Theisen

**Affiliations:** ^1^ Department for Congenital Disorders, Statens Serum Institut (SSI), Copenhagen, Denmark; ^2^ Centre for Translational Medicine and Parasitology at Department of Immunology and Microbiology, University of Copenhagen, Copenhagen, Denmark; ^3^ Laboratory of Malaria and Vector Research, National Institute of Allergy and Infectious Diseases, National Institutes of Health, Rockville, MD, United States; ^4^ Department of Molecular Microbiology and Immunology, Malaria Research Institute, Johns Hopkins Bloomberg School of Public Health, Baltimore, MD, United States; ^5^ Groupe de Recherche Action en Santé (GRAS), Ouagadougou, Burkina Faso; ^6^ Malaria Research and Training Center, Mali- National Institute of Allergy and Infectious Diseases International Center for Excellence in Research, University of Sciences, Techniques and Technologies of Bamako, Bamako, Mali

**Keywords:** Malaria, Circumsporozoite protein, CSP, vaccine, antibodies, clinical trial, Matrix-M

## Abstract

**Introduction:**

ProC6C is a multi-stage malaria vaccine which includes *Plasmodium falciparum* Circumsporozoite Protein (PfCSP), Pfs48/45 and Pfs230 sequences, designed to elicit functional antibodies that prevent sporozoite invasion of human hepatocytes (PfCSP) and parasite development in mosquitoes (Pfs48/45 and Pfs230). ProC6C formulated on Alhydrogel was evaluated in combination with Matrix-M in a Phase 1 trial in Burkina Faso. The PfCSP antibody responses were assessed for magnitude, specificity, avidity and functionality. These results compliment the prior reported safety and tolerability of ProC6C as well as the transmission reducing activity of ProC6C.

**Methods:**

The PfCSP response of ProC6C in Burkinabes in the Phase 1 trial (PACTR202201848463189) was profiled through the three vaccine administrations of 100 µg protein on Alhydrogel^®^ alone (AlOH) or combined with 50 µg Matrix-M™ adjuvant (AlOH/Matrix-M). Serology was completed against full-length PfCSP and major/minor repeat peptides using antibody equivalence to PfCSP monoclonal antibodies (mAb 311, mAb 317 and mAb L9). Comparison of the ProC6C responses were made to those that received RTS,S/AS01 in a study conducted in Thailand. Bio-Layer Interferometry was further used to determine antibody avidity. The human IgG was subsequently purified, pooled, and evaluated in a mouse sporozoite challenge model to determine functionality.

**Results:**

A single administration of ProC6C-AlOH/Matrix-M seroconverted 19 of 20 volunteers against PfCSP and significantly enhanced antibody titers to major and minor repeats (and present through D180). At D70, ProC6C-AlOH/Matrix-M PfCSP antibodies were found to be similar to responder pools generated from Thai adults receiving RTS,S/AS01. Additionally, ProC6C antibodies were found to be competitive to established PfCSP antibodies such as mAb 317 and mAb L9. The purified and pooled IgG from human volunteers, used in a passive transfer mouse sporozoite challenge model, showed a median of 50% inhibition (P=0.0058). ProC6C PfCSP antibodies were functional in this *in vivo* assessment and consistent with inhibition seen by other Circumsporozoite vaccines in this model.

**Discussion:**

This analysis supports continued investigation of the antibody responses elicited by the ProC6C multi-stage malaria vaccine. This Phase 1 clinical trial demonstrated the short PfCSP sequence included in ProC6C can induce significant PfCSP antibodies in humans, which importantly were determined to be functional.

## 1 Introduction

*Plasmodium falciparum* malaria is a parasitic infection with multiple developmental stages in mosquito and human hosts. Vaccines that target parasite development in the mosquito aim to reduce transmission of malaria parasites from infected to susceptible individuals, thereby reducing the number of secondary infections. Vaccines that target the sporozoite stage aim to provide direct protection against primary infections. The main target for such anti-infection vaccines is the *P. falciparum* Circumsporozoite protein (PfCSP), which is the immune dominant antigen on the sporozoite surface and essential for hepatocyte cell invasion (1, 2). PfCSP contains an immune dominant central repeat region, which is composed of 35-41 copies of NANP (3) and up to four copies of NVDP (4). The importance of this region is supported by *in vivo* data demonstrating that potent monoclonal antibodies (mAbs) mAb 317 (major repeat), mAb 311 (major-repeat), and mAb L9 (minor repeat) provided protection against an experimental challenge with transgenic *Plasmodium berghei* (*Pb*) sporozoites expressing full-length PfCSP (5, 6). This experimental challenge model is also supported by demonstrated protection through active vaccination of malaria vaccine candidates (7, 8). As the main epitope for antibodies, the NANP repeat sequence has been a major target for vaccine development including RTS,S/AS01 and R21/Matrix-M (9, 10). A phase 3 clinical trial of RTS,S/AS01 across eleven sub-Saharan African sites have demonstrated a vaccine efficacy (VE) against clinical malaria of ~ 27% in infants aged 6–12 weeks and ~ 46% in children aged 5–17 months (11). VE was correlated with the anti-CSP IgG antibody titer, which was initially high, but rapidly declined during the first four months post vaccination (10). While the exact reason for this short-lived antibody response is unknown, it has been proposed that the high number of NANP repeats may serve as an immune-escape mechanism by facilitating antibody homeotypic interactions, thereby limiting B-cell affinity maturation and long-term immunity (12–14). Whether epitopes in the central repeat region may elicit high levels of functional antibodies in humans when presented at a lower valency is not known.

**Figure 1:**
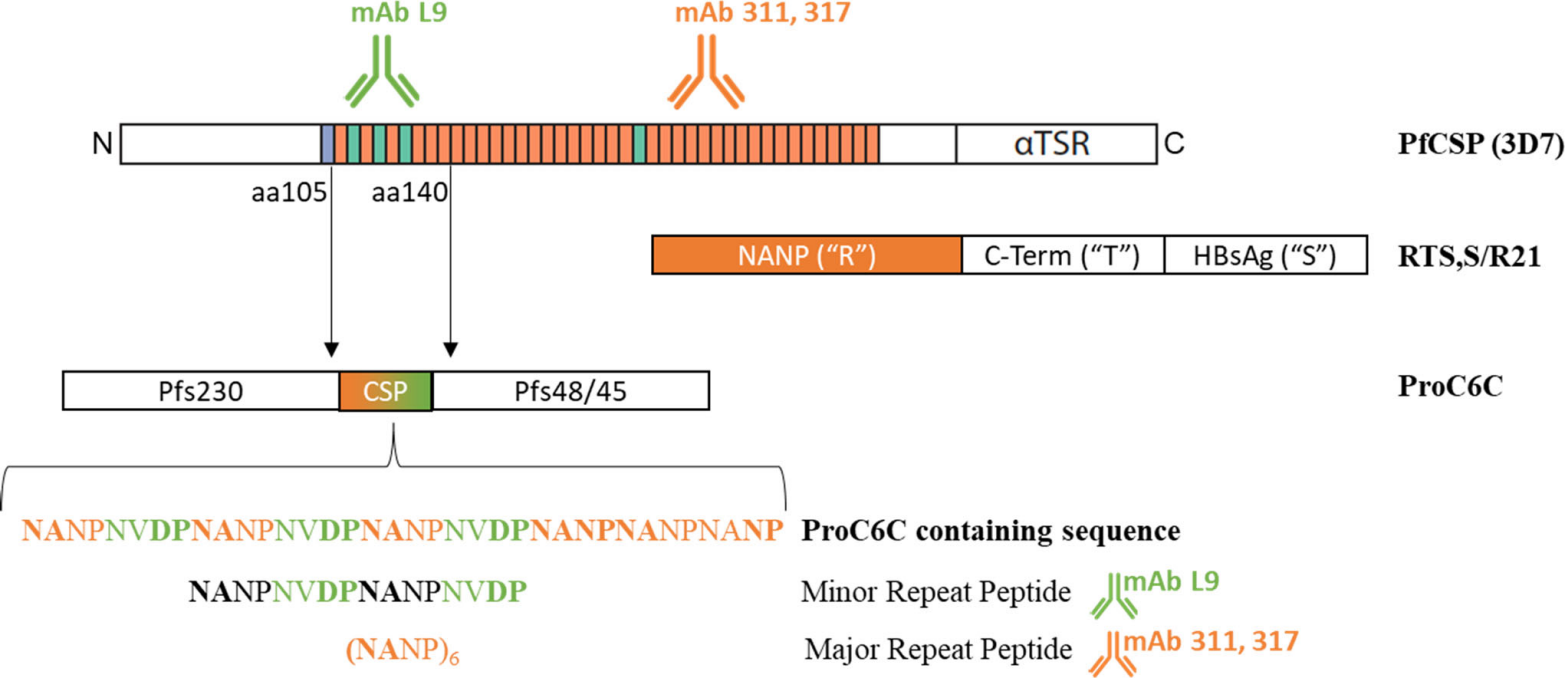
ProC6C is designed to encompass three protein domains: Two transmission-blocking vaccine domains Pfs230 and Pfs48/45 joined through a CSP sequence. The CSP sequence includes both central and minor repeats from the CSP sequence encompassing aa105-140 of the native 3D7 PfCSP sequence. In contrast, the RTS,S and R21 vaccines are based on the central repeat and C-terminus of the native CSP molecule (“R” and “T” respectively) fused with the Hepatitis B virus surface antigen “S”. The monoclonal antibody mAb L9 is reactive to the minor repeat sequence (green) and mAb 317 and mAb 311 to the major central repeats (orange). The minor and major repeat peptides used in the analysis are indicated.

Recently, we have designed a vaccine antigen, ProC6C (**Figure 1**) where a short PfCSP sequence composed of six copies of NANP and three copies of NVDP was inserted between an unstructured domain (Pro) from Pfs230 and a structured domain (6C) of Pfs48/45 (15). Encompassing three total protein domains, ProC6C aims to be a multi-stage malaria vaccine eliciting antibodies that prevent transmission and block individual infection, through a single administered protein. In preclinical models, ProC6C elicited high titers of transmission blocking (TB) antibodies together with antibodies that block hepatocyte invasion by sporozoites (15). ProC6C adjuvanted with Alhydrogel (AlOH) with and without Matrix-M™ has completed Phase I clinical evaluation in Burkina Faso [PACTR202201848463189 (16)] and Mali [ISRCTN13649456 (17)]. All formulations were found to be safe and well tolerated (16). Prior analysis of the Phase I clinical trial in Burkina Faso demonstrated that ProC6C-AlOH/Matrix-M was superior in immunogenicity to the vaccine immunogen (ProC6C) and also elicited a transmission reducing activity of >80% in 65% (13/20) of the volunteers. This transmission reducing activity correlated to the Pfs48/45 antibody titers (16).

Here, we describe the antibody responses against PfCSP elicited by the ProC6C vaccine in terms of magnitude, specificity, avidity, and functionality from clinical trial volunteers from the Burkina Faso trial. We found ProC6C elicited PfCSP IgG antibodies to both the major and minor repeats, and those antibodies were competitive to established PfCSP monoclonal antibodies mAb 317 and mAb L9. Total IgG from those same volunteers were functional in preventing sporozoite invasion of the liver in a transgenic challenge model.

## 2 Materials and methods

### 2.1 Study design

The study (“TBVax1”) was a randomized, staggered, adjuvant-selection, dose-escalation phase 1 clinical trial conducted in the Sabou health district of Burkina Faso in healthy adults, aged 20 to 45 years (16). Participants (n=20 enrolled to each group) received intramuscularly 100 μg ProC6C-AlOH (Statens Serum Institut, Denmark) alone, called “G2C” in this manuscript, or supplemented with 50 μg Matrix-M™ adjuvant (Novavax, Sweden) called “G2D” or a placebo Hepatitis B vaccine, Euvax B (LG Chem, The Republic of Korea) called “G2E”. A series of three immunizations were given on days (0, 28, 56) and sera collected on days 0, 14, 28, 42, 56, 70, 140 and 180 (16). The safety, tolerability and transmission blocking activity elicited by ProC6C in this trial was previously reported (16).

### 2.2 Study sample availability

The present analysis included participants from the 100 µg ProC6C-AlOH (G2C) and 100 µg ProC6C-AlOH/Matrix-M (G2D) and the Hepatitis B (HepB, G2E) control groups. For the primary endpoint of D70, all twenty samples were available from each the ProC6C-AlOH and ProC6C-AlOH/Matrix-M vaccine groups, while 19 out of 20 samples were available from the control group (G2E) due to withdrawal of consent (n=1). For durability (D140 and D180) due loss to follow-up, sample numbers included n=18; n=20, and n=19 for G2C, G2D, and G2E respectively. Paired analysis (D70 and D180) utilized only individuals with all timepoints available. Study samples analyzed from TBVax1 (PACTR202201848463189) within the studies reported here are summarized in **Table 1**.

**Table 1:** TBVax1 Sample Availability.

Group	Enrolled (n)	Vaccine	Antigen Dose (μg)	Matrix-M Dose (μg)	D70 Samples Available (n)	D180 Samples Available (n)
2C	20	ProC6C-AlOH	100	–	20	18
2D	20	ProC6C-AlOH/Matrix-M	100	50	20	20
2E	20	Placebo/Hepatitis B	20	–	19	19

### 2.3 RTS,S/AS01 comparative sample pools

Immune serum pools from RTS,S/AS01 immunized individuals were provided by Mahidol Oxford Tropical Medicine Research Unit (MORU)/Mahidol University, Bangkok, Thailand through PATH. Individuals in this Phase 2 open-label, randomized, controlled study in health, adult Thai volunteers received up to three doses of RTS,S/AS01 one-month apart with the primary immunogenicity read-out at three months post primary vaccination (or one-month post last-dose) (18). Serum samples were pooled from treatment groups 1,2,3 and 5 (18) based on PfCSP antibody titers. Antibody titers used were from prior analysis as reported (18) and utilized titers of 100-1000, 1001-10,000, and above 10,001 to generate three pools: low, medium and high respectively. Samples used in the pool (2 mL from each individual and 19-20 individuals for each pool) were based strictly on titer and included samples from one month post first dose, one month post second-dose and one moth post third/last dose. Pools were de-identified, aliquoted into 0.5 mL aliquots and frozen at -80°C for use in the analysis reported here.

### 2.4 Enzyme-linked immunosorbent assays

#### 2.4.1 PfCSP direct ELISA

Vaccine-specific IgG antibody levels were determined by ELISA as previously described (15). Briefly, plates were coated with 0.5 μg/mL PfCSP4/38 (19), a full-length PfCSP recombinant protein containing 4 NVDP and 38 NANP repeats. Serum was analyzed at various dilutions and antibody concentration was normalized against mAb 311 (6) and developed using HRP polyclonal Rabbit anti-human IgG (Agilent, Denmark) at 1:3000 and TMB substrate with 100 µl H_2_SO_4_.

#### 2.4.2 Major and minor repeat direct ELISA

Epitope-specific anti-PfCSP antibodies were also determined by ELISA as described for PfCSP4/38, however, the plates were pre-coated with 1μg/mL neutravidin prior to coating with 0.5 μg/mL of either major repeat (NANP)_6_ or minor repeat (NANPNVDPNANPNVDP) antigens (**Figure 1**) that were biotinylated (ProteoGenixSAS, France). The major and minor specific antibody concentrations were normalized against mAb 311 and mAb L9 respectively.

#### 2.4.3 Proportional antibody responses by competition ELISA

The relative proportions of antibodies to repeat regions were obtained by a peptide-competition ELISA. Individual serum samples were incubated with major repeat peptide (6 µg), a mixture of major + minor repeat peptides (6 µg each) or PfCSP4/38 (6 µg) for 1 hour at room temperature (RT) on a shaker. Serum samples, with or without peptide/protein (blank) were added to PfCSP4/38 coated ELISA plates for 1 hour at RT with gentle shaking and optical density (OD) measured as previously described. As expected, prior incubation of sera with PfCSP4/38 reduced the OD signal to baseline. Absolute reduction was calculated as: (OD _(peptide/protein)_/OD _blank_) x 100%. Absolute reduction obtained with PfCSP4/38 was set at 100%. To correct for plate-plate and day-day variations, relative reductions were calculated using PfCSP4/38 reduction as the reference. Relative peptide reduction was calculated using the formula: (Absolute reduction _peptide_/Absolute reduction _PfCSP4/38_) X 100%. The anti-CSP domain proportional responses were obtained by the following formular: Minor repeat = Absolute reduction _(major + minor)_ – Absolute reduction _major_ and Non-repeat domain = Absolute reduction _PfCSP4/38_ - Absolute reduction _(major + minor)_.

#### 2.4.4 Monoclonal antibody competition ELISA

The mAb 317 and mAb L9 were utilized in a competition ELISA to determine monoclonal-like antibodies elicited by ProC6C and present in vaccinees. Serum pools at D0, D70 and D180 at different dilutions were incubated with PfCSP4/38 (12.5 ng/mL for 317 and 6.25 ng/mL for L9) for 1 hour. Following incubation, samples were added to plates pre-coated with 317 (2 µg/mL) and L9 (5 µg/mL) and incubated at room temperature for 1 hour with gentle shaking after which the plates were washed and developed using anti-His IgG-HRP (1:3000) to detect bound PfCSP4/38 and TMB substrate with 100 µl H_2_SO_4_.

### 2.5 Biolayer interferometryavidity assay

BLI measurements were performed as described (20) with modifications using ForteBio Octet RED96e instruments and ForteBio biosensors to measure the ProC6C vaccine-induced serum antibody binding response and the rate of dissociation after interaction with PfCSP peptides (major and minor repeats). ProC6C-AlOH/Matrix-M group sera were tested for peptides binding in triplicate at 1:50 dilution. The biotinylated peptides: major (10 µg/mL) and minor (0.625 µg/mL) peptides were loaded onto streptavidin (SA) coated sensors to monitor dissociation rates. To subtract binding due to non-specific interaction, a reference sample well (sensor with ligand but no analyte) and a reference sensor (sensor with no ligand against an analyte) were measured and subtracted from their specific interactions to obtain the binding response and dissociation rate. For assay qualification, anti-CSP mAbs (mAb 317 and mAb L9) at various concentration (16, 8, 4, 2, 1, 0.5 µg/mL) were tested against major and minor repeats respectively to obtain Ab association (k_a_), dissociation (k_d_) and equilibrium (K_D_) rate constant values and compared to that of previously published (**Supplementary Figure S1**). Kinetics were performed at 25°C and data analyses were done using Octet analysis studio 12.2 and GraphPad Prism 9.3.1.

### 2.6 Functional assessment of purified IgG through the liver invasion challenge model

Total IgG was purified from D70 sera (100 µg ProC6C-AlOH/Matrix-M, G2D group) individually using Protein G columns. Based on the ELISA titers against PfCSP4/38, IgGs were pooled (Hi from n=6 and Mid from n=7) along with Pooled IgG (D70, HepB, G2E group). The three pooled IgGs were adjusted to 50 mg/mL in 1xPBS.

Purified IgG was assessed in a transgenic *P. berghei* sporozoite mouse challenge model as described (21–23). Six-to-eight weeks old C57/BL/6 female mice received 10 mg/mouse of the pooled IgG via intravenous (iv) injection (n=5). As positive and negative controls, 5 mice received 100 μg/mouse of mAb 317, and 5 were untreated (naïve). Two hours after treatment, mice were challenged with 2x10^3^ chimeric *P. berghei* sporozoites expressing PfCSP by iv. Forty-two hours after challenge, mice were injected with 100 μL of D-Luciferin (30 mg/mL), anesthetized with isoflurane and imaged with the IVIS spectrum Imager to measure the bioluminescence expressed by the chimeric parasites. The mouse study was approved by the Johns Hopkins ACUC, protocol number MO21H417.

### 2.7 Statistical analysis

Data was processed using Quant Assay for Windows (Version 0.7.1.4) and GraphPad Prism Version 8.3.0. For ELISA data, all analysis were conducted using log-transformed ELISA titers. The ELISA data among different groups at the same time point were compared by a One-way ANOVA followed by Tukey’s multiple comparisons test. Comparisons across multiple times were calculated by a repeated-measures One-way ANOVA followed by Tukey’s multiple comparisons test. For correlation analysis, a Pearson test was utilized. The other analyses were conducted by non-parametric tests (Kruskal-Wallis test followed by Dunn’s multiple comparison tests for non-paired data set, and Friedman test followed by Dunn’s multiple comparison tests for paired data set). P < 0.05 was considered significant.

## 3 Results

### 3.1 ProC6C elicit high titer antibodies against full-length PfCSP in humans

**Figure 2:**
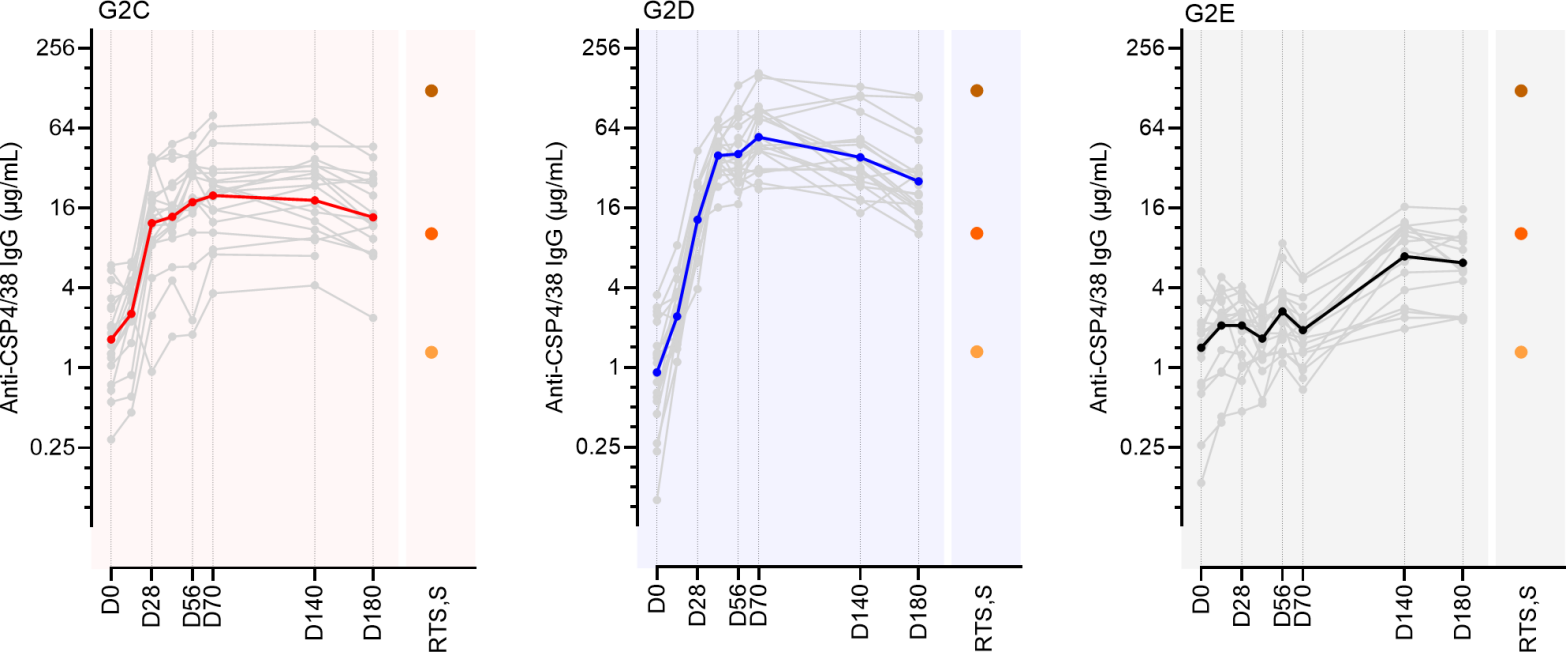
Antibody reactivity to full length CSP, PfCSP4/38, in individuals immunized with ProC6C-AlOH (red, G2C), ProC6C-AlOH/Matrix-M (blue, G2D) or a control vaccine (black, G2E). Anti-CSP4/38 IgG levels are given as mAb 311 equivalence (µg/mL). Geometric mean titer (GMT) for each group represented by solid colored line, with individuals shown by gray line. For comparison we show IgG antibody levels to 3 pools of serum samples from high (dark orange), medium (orange), and low (light orange) responding Thai adults vaccinated with RTS,S/AS01 (18).

ProC6C (**Figure 1**) contains protein sequences derived from Pfs230 (“Pro”), PfCSP (“C”) and Pfs48/45 (“6C”) (16). Previously we reported antibody responses against the ProC6C vaccine antigen, but PfCSP antibody responses were not initially evaluated (16). Here, we determined specific antibodies against a full-length PfCSP recombinant protein (PfCSP4/38) generated after vaccination at each timepoint in volunteers receiving 100 µg ProC6C-AlOH (G2C) and ProC6C-AlOH/Matrix-M (G2D), or the Hepatitis B (G2E) control vaccine (**Figure 2**). A single administration of ProC6C-AlOH or ProC6C-AlOH/Matrix-M seroconverted 17/19 and 19/20 volunteers respectively, with seroconversion defined as mean + 2 standard deviation of log-transformed titer at baseline. After 3 vaccinations, D70 PfCSP-specific geometric mean titer (GMT) increased to 20.0 (95% confidence interval, 95%CI: 13.8-28.7) and 54.4 (41.8-70.9) for groups G2C and G2D, respectively (**Figure 2**). The GMT did not increase in the control group (D0, 1.3 (0.9-2.0); D70, 1.8 (1.4-2.3), although due to natural exposure PfCSP antibodies were present. At D70, group G2D showed significantly higher antibody titer compared to G2C (P=<0.0001) and G2E (P=<0.0001) after correction for multiple comparisons, thus confirming the potent adjuvant activity of Matrix-M (**Figure 2**). The Hepatitis B (G2E) control vaccine group showed an increase in background PfCSP-specific titers due to natural transmission and exposure during the study conduct as detailed in (16). As expected, levels of PfCSP IgG decreased over time. At D180, GMT was 14.3 (9.7-20.9) and 25.3 (18.4-34.8) in groups G2C and G2D, respectively and 5.8 (4.2-8.2) in the control group (**Figure 2**).

To compare PfCSP IgG levels elicited by ProC6C with those elicited by an established malaria vaccine presenting NANP at a high valency, we also analyzed three pools of serum samples from high, medium, and low titer responses in Thai adults vaccinated with up to three doses of RTS,S/AS01 and sera collected one-month after vaccination (18). ProC6C-AlOH/Matrix-M vaccinated individuals induced PfCSP IgG responses comparable to and in the range of the mid and high titer RTS,S/AS01 pools at D70 and D180 (**Figure 2**).

### 3.2 ProC6C elicits IgG antibodies against the major and minor repeats of PfCSP

**Figure 3:**
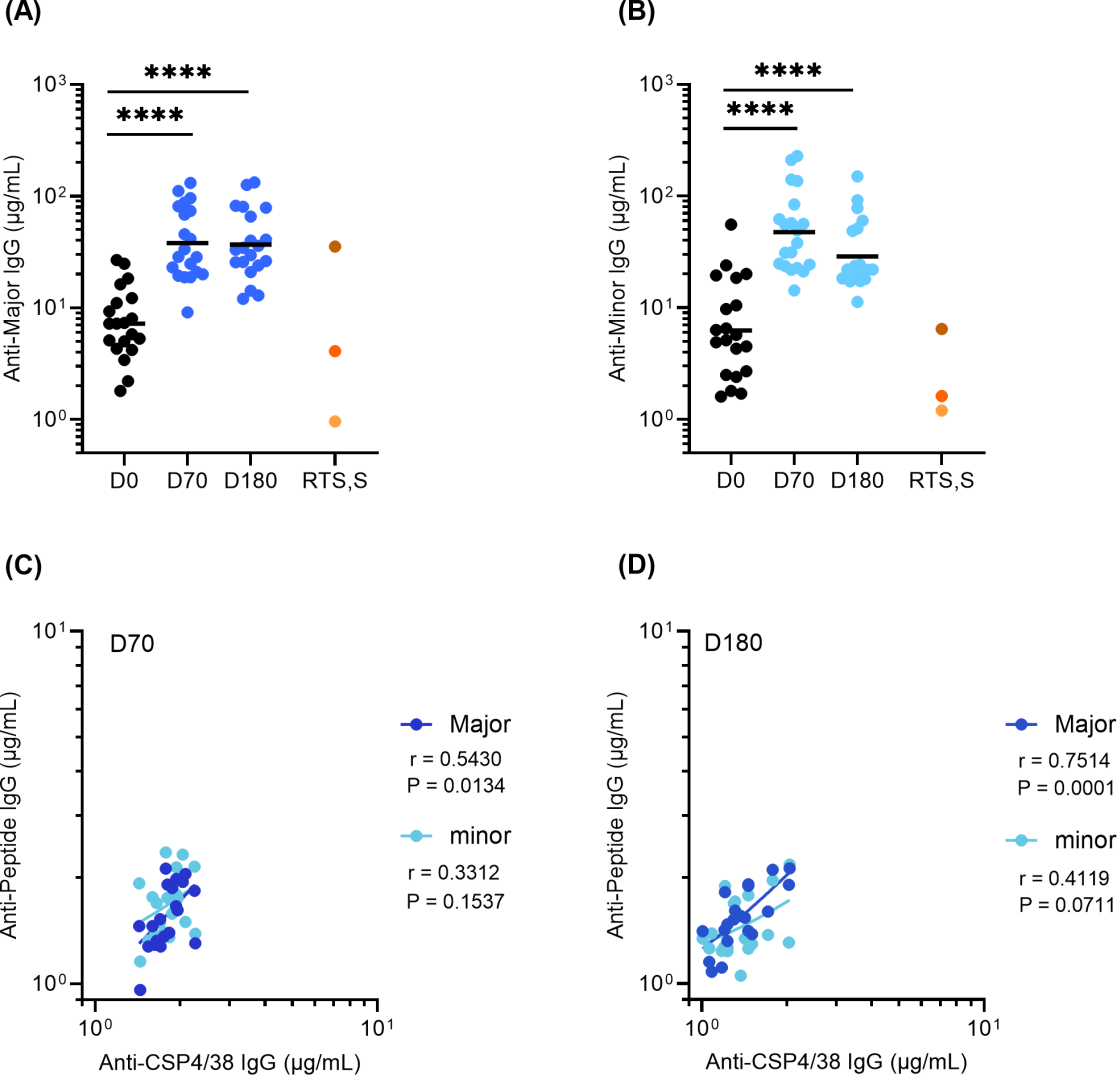
Major and Minor repeat antibodies elicited by ProC6C-AlOH/Matrix-M (G2D). IgG was evaluated by peptide ELISA using major repeat peptide: (NANP)_6_ and minor repeat peptide: NANPNVDPNANPNVDP as plate antigens. **(A)** Anti-major repeat IgG levels (D0, D70, D180) and **(B)** Anti-minor repeat IgG levels (D0, D70, D180) from individuals receiving three vaccinations of G2D on D0, D28, D56 are given as **(A)** 311 or **(B)** L9 equivalence (µg/mL). The GMT for each day is shown by solid line. Statistical significance is indicated between days by one-way ANOVA with Tukey’s multiple comparison test. ****P < 0.0001 RTS, S/AS01 pools as in **Figure 2** indicated by shades of orange circles. Anti-peptide IgG (Y axis) plotted against PfCSP4/38 IgG (X axis) for D70 **(C)** and D180 **(D)**. Pearson correlation coefficients (r) are shown for major (dark blue) and minor (light blue). Simple linear regression indicated by solid line respectively. Major and minor repeat antibodies elicited by Proc6C-AlOH (G2C) are shown in **Supplementary Figure S2**.

Given ProC6C-AlOH/Matrix-M (G2D) elicits a superior immune response than ProC6C-AlOH (G2C), subsequent analysis was focused on Group G2D (**Figure 3** and G2C results are reported in **Supplementary Figure S2**). The specificity of antibodies was measured using ELISA plates coated with the major and minor repeat peptides. At D70, the GMT of serum IgG increased to 38.0 (26.8-53.8) and 20.1 (13.8-29.3) against the major and minor repeats, respectively (**Figures 3A, B**). These antibodies remain consistent at D180 for the major repeat and decreased for the minor repeat. As expected, pre-existing PfCSP antibodies were present at D0, due to prior malaria exposure in this endemic adult population.

Further, the ProC6C-AlOH/Matrix-M antibody responses were evaluated in context against those achieved by RTS,S/AS01 through using three RTS,S/AS01 pools (**Figures 3A, B**). At D70 and D180, anti-Major repeat ProC6C-AlOH/Matrix-M IgG was comparable to that of the RTS,S/AS01 high responders (**Figure 3A**). In contrast, ProC6C-AlOH/Matrix-M elicited anti-minor IgG that was 3.6 (D70) and 1.7-fold (D180) higher than that of the RTS,S/AS01 high responders (**Figure 3B**). As noted above, given prior natural exposure in the Burkinabe adults, the D0 titers were equivalent to that of the RTS,S/AS01 (from an adult Thai population) medium pool (Major repeat) and high pool (Minor repeat).

The correlation between anti-peptide and full-length (anti-CSP4/38) IgG responses were also evaluated for D70 and D180 (**Figures 3C, D**, respectively). There is a positive correlation (P=0.0134, r=0.5430 for D70 and P=0.0001, r=0.7514 for D180) for the major repeat and a non-significant correlation (P=0.1537, r=0.3312 for D70 and P=0.0711, r=0.4119 for D180) for the minor repeat.

**Figure 4:**
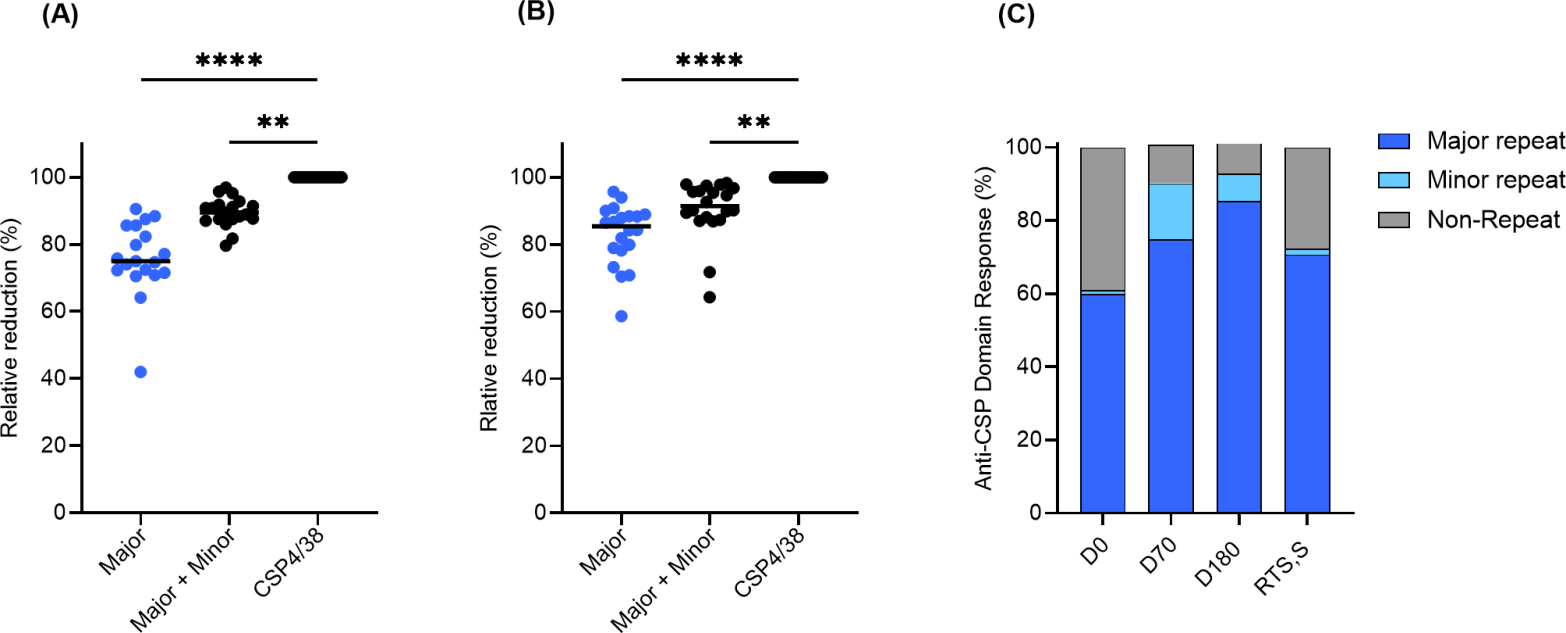
Relative proportion of PfCSP antibodies against major, minor and non-repeat domains determined by competition ELISA for ProC6C-AlOH/Matrix-M (G2D). D70 serum samples **(A)** and D180 serum samples **(B)** were incubated with saturating amounts (6 µg) of peptides representing major and minor peptides and full-length PfCSP (PfCSP4/38) as a reference (100%). ELISA signal obtained with incubation of PfCSP4/38 is first determined and set at 100% reduction. Signals are then compared to that obtained with either the major or major + minor peptides mixtures, indicating a relative proportion of antibody. Individual circles indicate reduction obtained in an individual serum sample. Statistical significance is indicated between antibody responses by Friedman test with a Dunn’s multiple comparisons, **P <0.01 and **** P < 0.0001. Relative proportion of PfCSP domain specific IgG **(C)** for D0, D70, D180 and RTS,S/AS01 (medium). The average minor domain response on each day was calculated by subtracting the major peptide reduction from the major + minor peptides reduction from panels A and B (D0 was determined from a pool generated from all individuals). The average non-repeat response was calculated by the residual reduction not accounted for from the major + minor peptide mixture.

Next, to determine the relative proportion of antibodies against the repeats, they were tested for their ability to inhibit the binding of serum antibodies to the full-length molecule (PfCSP4/38) which includes domains outside of the major and minor repeats. Saturating amounts of the major and minor repeat peptides were added to individual D70 and D180 serum samples from ProC6C-AlOH/Matrix-M (G2D) and subsequently, the effect on the binding to full-length PfCSP4/38 coated ELISA plates was determined. In parallel experiments, the effect of prior incubation with full-length PfCSP4/38 was determined as a reference (**Figure 4**). The binding of D70 serum antibodies to recombinant PfCSP4/38 was strongly reduced by prior incubation with the major peptide with a median relative reduction of 75.0% (95% CI;71.5-85.6). The addition of the minor repeat to the major (major + minor) resulted in a further 14.5% reduction representing minor epitope specific antibodies (**Figure 4A**). At D180, the relative reduction of the major peptide was 85.4% and minor 6.0% (**Figure 4B**). Taken together, the overall proportion of antibody response is shown prior to vaccination (D0) and after vaccination at D70 and D180 (**Figure 4C**). The non-repeat reactivity accounted for 10.5% (95% CI; 8.2-12.5) of the anti-CSP reactivity at D70 and 8.6% (95% CI; 4.1-8.7) at D180. While the anti-major repeat IgG is dominant before and after vaccination, the ProC6C vaccine enhances the relative proportions of anti-minor repeat IgG, that decreases over time. In comparison, the RTS,S/AS01 responder pool (medium pool) generated a predominant major-repeat response (~75%) as expected (**Figure 4C**).

### 3.3 ProC6C elicits antibodies with similar specificities as functional PfCSP monoclonal antibodies

**Figure 5:**
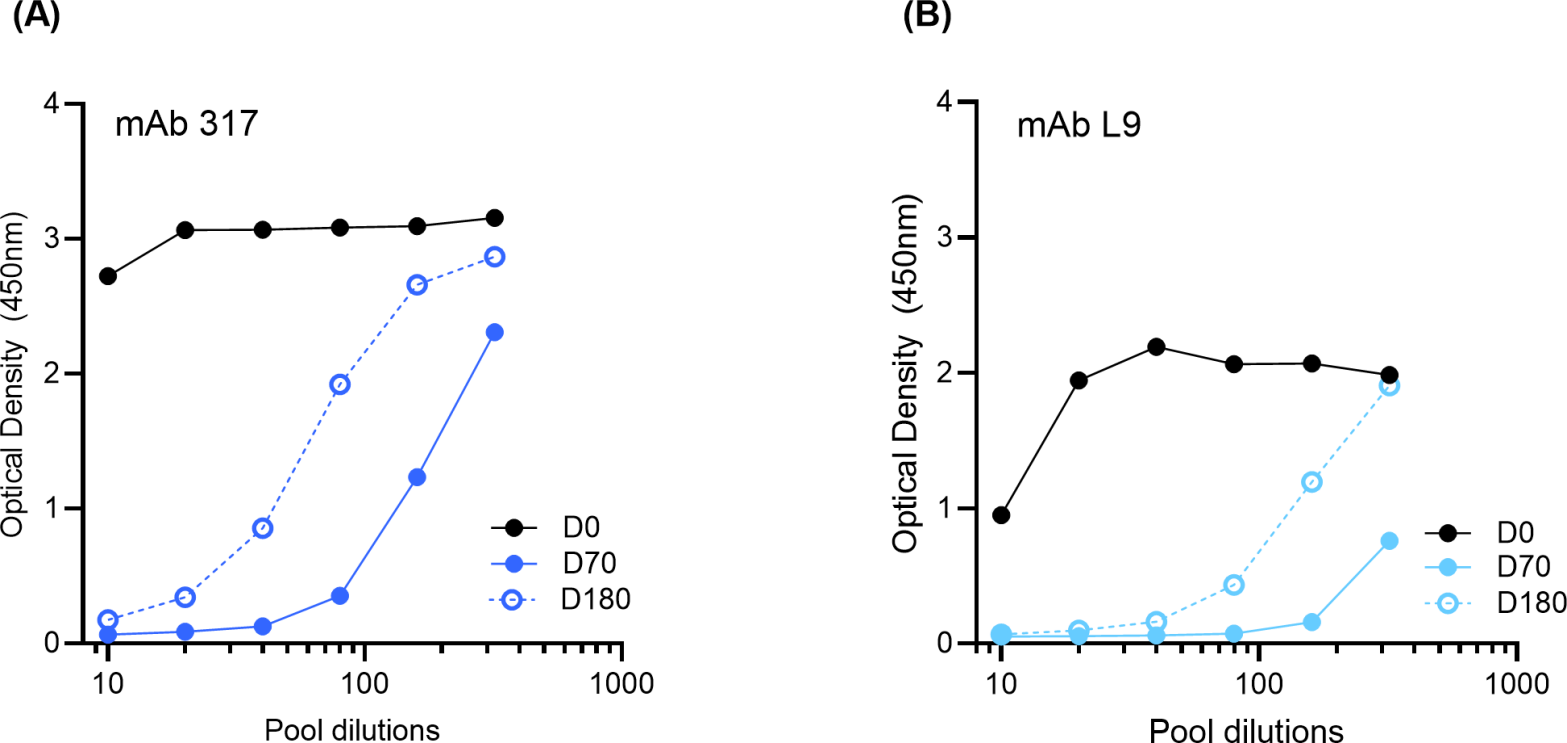
ProC6C-AlOH/Matrix-M elicited antibodies that competed with mAb 317 and mAb L9. PfCSP4/38 was incubated with serial dilutions of ProC6C-AlOH/Matrix-M (G2D) anti-sera from D0, D70 or D180, then the mixtures were added to ELISA plates coated with monoclonal antibodies that react to major and minor antibodies: mAB 317 **(A)** or mAb L9 **(B)** respectively.

To determine if the major and minor repeat antibodies induced by ProC6C-AlOH/Matrix-M (G2D) were similar to those of known potent PfCSP monoclonal antibodies, major repeat mAb 317 and minor repeat mAb L9 were used in a competition ELISA (**Figure 5**). Serial dilutions of sera pools from D0, D70 and D180 of ProC6C-AlOH/Matrix-M vaccinated individuals were pre-incubated with PfCSP4/38, followed by addition to ELISA plates pre-coated with mAb 317 (**Figure 5A**) or mAb L9 (**Figure 5B**). In the analyses, both monoclonal antibodies bound to PfCSP4/38 in the presence of D0 sera, but decrease upon addition of D70 or D180 sera, indicating competition for the binding sites of mAb 317 (**Figure 5A**) and mAb L9 (**Figure 5B**).

### 3.4 Avidity of repeat-specific antibodies in ProC6C vaccinated individuals

**Figure 6:**
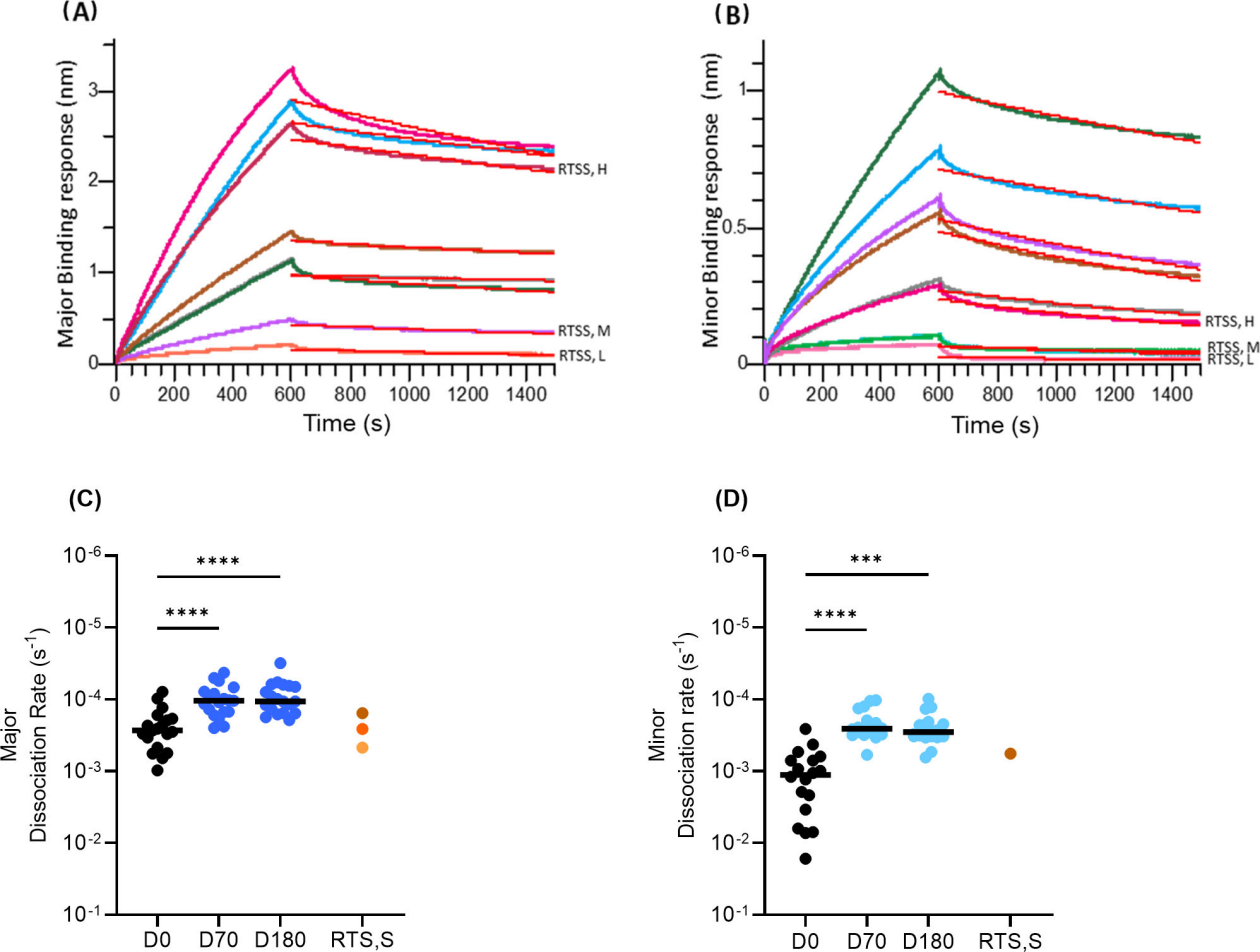
Avidity of anti-CSP antibodies over time determined by dissociation rates of IgG binding to **(A)** major and **(B)** minor-repeat peptides determined by BLI. Dissociation rates to major **(C)** and minor **(D)** repeat peptides at D0, D70 and D180. Individual samples indicated by closed circles as a mean of triplicates. The median for each day is shown by solid line. Statistical significance is indicated between days by Friedman test with a Dunn’s multiple comparisons, ***P < 0.001 and ****P < 0.0001. RTS,S/AS01 pools as in **Figure 2** and **Figure 3** indicated by orange circles as before. For **(D)**, only the RTS,S/AS01 (high) result is shown, as there was no detectable binding with the other two RTS,S/AS01 pools.

To assess the change in avidity over time, we determined dissociation rates of individual serum IgG binding to the major and minor-repeat peptides. The sensorgram obtained for major and minor repeats using BLI are shown (**Figures 6A, B**). There was a significant decrease in median dissociation rates of serum IgG binding to both the major (**Figure 6C**) and minor (**Figure 6D**) peptides between D0, D70 and D180 in group ProC6C-AlOH/Matrix-M (G2D) (P<0.0001 against major for D70 and D180, **Figure 6C**; P<0001 (D70) and P=0.0007 (D180) against minor, **Figure 6D**). For both major and minor peptides there was no difference (P>0.9999) between D70 and D180 avidity, indicating avidity did not change over time.

Anti-major repeat of ProC6C-AlOH/Matrix-M IgG BLI responses were comparable to that of the RTS, S/AS01 high responders at D70 (**Supplementary Figure S3A**). However, response in D180 was 3.3-fold higher than the RTS,S/AS01 medium responders. ProC6C-AlOH IgG on the other hand showed a 2.1-fold and 1.6-fold higher response compared to RTS,S/AS01 medium responders at D70 D180 respectively. Expectedly, RTS,S/AS01 samples elicited no anti-minor IgG except the high responders pool which showed minimal levels and likely due to background exposure (**Supplementary Figure S3B**). Further BLI responses confirmed that ProC6C-AlOH/Matrix-M was superior to the ProC6C-AlOH against both the major and minor peptides (**Supplementary Figure S3**). Moreover, the major and minor repeat responses correlated well (P<0.0001, r > 0.7 for D70 and D180) with corresponding ELISA-titers confirming the antigenic conformation of the peptides in the BLI assay (**Supplementary Figure S4**).

### 3.5 Vaccine-specific ProC6C human IgG protect mice from intravenous challenge with *Plasmodium*

**Figure 7:**
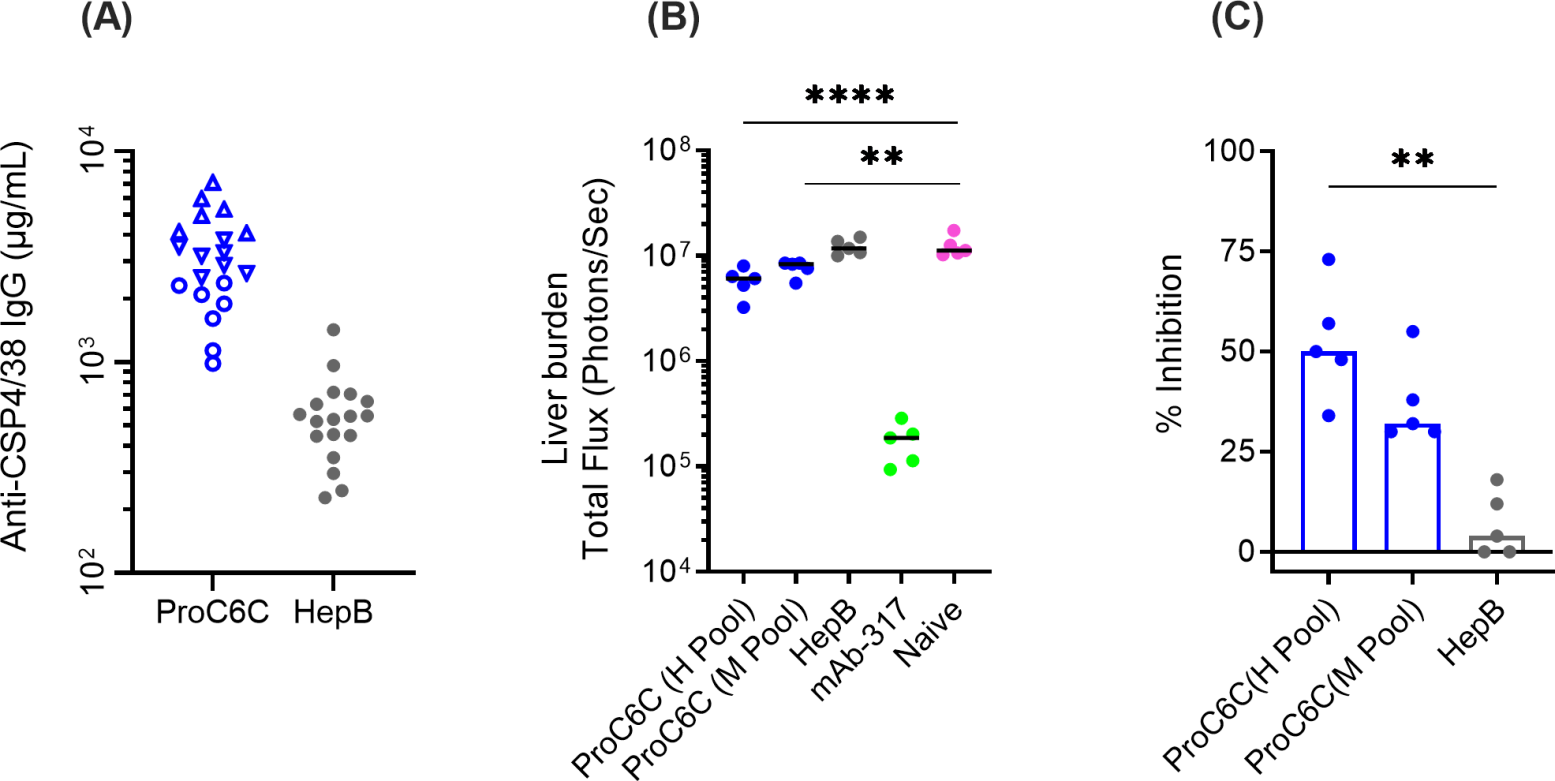
Functionality activity of human IgG from ProC6C-AlOH/Matrix-M vaccinated volunteers. **(A)** The presence of PfCSP IgG (at 50 mg/mL) was determined in purified IgG from individuals immunized with ProC6C-AlOH/Matrix-M (G2D) and Hepatitis B (HepB, G2E) by ELISA using PfCSP4/38 as the plate antigen. Titers were used to generate pools (High titer, open up triangle, Mid titer, open down triangle and remaining individuals closed circle). Pools were then analyzed for mAb 311 equivalence and reported as follows: High titer pool (H pool) 555 µg/mL; Mid titer pool (M Pool) 23 µg/mL and HepB 5 µg/m. Purified IgG pools: High, Mid, and HepB (10 mg IgG/mouse, 200 μL) were analyzed in the transgenic sporozoite challenge model to assess liver burden alongside mAb 317 as positive control and reported as total flux **(B)** and percent inhibition **(C)** compared to naïve control (untreated). Median and individual values are shown. Statistical significance is indicated between treatment by One-way ANOVA and Kruskal-Wallis test followed by Dunn’s multiple comparison for 7B and 7C respectively. **P < 0.01 and ****P < 0.0001.

To examine whether human PfCSP IgG antibodies elicited after vaccination might confer protection against sporozoite-infection, we used a well-established model for assessing vaccine efficacy. In this model, mice were passively immunized with purified IgG from trial participants, then challenged with transgenic *Plasmodium berghei* (*Pb*) where the native CSP gene has been replaced with full-length PfCSP and a luciferase reporter (*Pb*-*Pf*CSP-*Luc*) (21). Following intravenous injection of sporozoites, parasite burden was quantified by measuring luciferase levels in the liver. Total IgG was purified from volunteers vaccinated with ProC6C-AlOH/Matrix-M (G2D) or HepB (G2E). Based on PfCSP antibody titers against the PfCSP4/38 protein, pools of IgG, ProC6C-Hi and ProC6C-Mid were generated together with an IgG pool from the HepB group (**Figure 7A**).

Groups of 5 mice were injected intravenously (iv) with 10 mg (200 μL)/mouse of the respective IgG pools and two hours later iv challenged with 2x10^3^ chimeric sporozoites (**Figure 7B**). Control mice were untreated (naïve) or treated with mAb 317 (targeting the major central repeat) as negative and positive controls. As expected, all mice in the mAb 317 group showed strong reduction in liver burden (median of 98% inhibition). When the Flux signals were compared among naïve and IgG injected groups, there was insignificant difference between HepB and naïve groups (4% inhibition, P>0.999). On the other hand, ProC6C-Hi significantly reduced parasite liver burden by 50% (P=0.0058). ProC6C-Mid demonstrated 32% inhibition, but it did not reach significance (P=0.0852) (**Figure 7C**). Taken together, these results demonstrate that ProC6C-AlOH/Matrix-M induced functional antibodies in humans as evaluated in this model.

## 4 Discussion

In summary we showed that the multi-stage vaccine candidate ProC6C-AlOH/Matrix-M elicited functional anti-infection antibodies against the malarial sporozoite stage. Levels of PfCSP-specific IgG antibodies from Burkinabe adults receiving ProC6C (0, 1, and 2 month schedule) were comparable to those from a Thai adult population that received RTS,S/AS01. Previous work reported that ProC6C-AlOH/Matrix-M following the same delivery schedule elicited high levels of functional antibodies against *P. falciparum* transmission-stages in Burkinabé adults, but whether these vaccine-specific antibodies were also functional against the sporozoite-stage was not known (16). Here, we report that the same Burkinabé adults elicited specific antibodies against both the major (NANP) and minor (NVDP) repeats of *P. falciparum* CSP with the major repeat accounting for approximately 75% of the anti-CSP IgG at D70 (14 days after 3^rd^ vaccination) as determined by an ELISA-based competition assay. The remaining 15% and 10% of the anti-CSP IgG was directed against minor-repeat and non-repeat sequences, respectively.

At D70, anti-PfCSP responses by ProC6C-AlOH/Matrix-M were comparable to the Thai population receiving RTS,S/AS01 (containing 19 copies of NANP). This suggests it may be possible to elicit high PfCSP antibody titers with an adjuvanted vaccine of low valency such as ProC6C-AlOH/Matrix-M (containing six copies of NANP). While repeat regions of malarial antigens in general are immune-dominant and generate strong anti-parasite antibody responses (24), it has been speculated that the high number of NANP repeats may also serve as an immune-escape mechanism by suppressing immune response against other more “protective” epitopes on the sporozoite surface (25–27). Further, the high number of NANP repeats in PfCSP may lead to structures facilitating high affinity interactions between anti-CSP IgG antibodies and their target epitopes thereby favoring the IGHV3-33/IGKV1-5 gene combination found in many of the most potent PfCSP-specific mAbs (12, 14, 28–30). Such high-affinity interactions may limit B-cell affinity maturation in germinal centers and the induction of long-lived antibody responses (12–14). Interestingly, we found specific NANP-responses did not decrease significantly between days 70 and 180 suggesting that vaccine-specific responses might be qualitatively different from those of high-valency vaccine antigens. Whether the lower valency of PfCSP repeats in ProC6C favors other heavy/light chain gene combination and the longevity of antibody responses remains to be investigated.

Our finding ProC6C-AlOH/Matrix-M elicited higher proportion of IgG against the minor repeat compared to RTS,S/AS01 was not surprising as ProC6C contains three NVDP repeat units. While the major repeat region is a well-established target for protective immunity afforded by RTS,S/AS01 (10) little is known about the role of antibodies against the minor repeats in protective immunity and additionally there is potential for major/minor cross reactivity (5). Recently, the mAb L9 against the minor repeat was identified as a highly potent anti-PfCSP mAb, which confer sterilizing immunity against Controlled Human Malaria (CHMI) in humans (31). Encouragingly, we found that ProC6C-AlOH/Matrix-M elicited anti-CSP IgG antibodies with similar specificity as L9 confirming the ability of ProC6C to promote antibodies against the minor repeats. Since vaccination aims to increase both concentration and avidity of specific antibodies, the avidity of serum IgG was measured by BLI using the minor and major repeat peptides as probes. We found the avidity of antibodies against the minor (and major) repeat increased 11-fold between days 0 and 70 and remained 9.5-fold higher at day 180.

To examine whether human anti-ProC6C antibodies might confer protection against sporozoite infection, we used the transgenic rodent *P. berghei* (*Pb*) model for assessing vaccine efficacy against liver infection (7, 22). In this model, passive transfer of human IgG with high PfCSP IgG concentration significantly reduced liver infection by 50% (P=0.0058). In contrast, pooled IgG from the control group and a pool of IgG with a low PfCSP IgG concentration had no such activity, demonstrating that the protective effect was vaccine-specific and dose-dependent. Limitations in the RTS,S sera pools did allow for purified IgG to be included, however passive transfer from individuals immunized with RTS,S sera in this model demonstrates similar levels of inhibition (7). While the challenge model (*Pb*-*Pf*CSP-*Luc)* remains the most advanced preclinical rodent model to measure vaccine efficacy in a preclinical setting, there is an inherent risk that this model may not fully replicate the human situation. However, this remains the current best-characterized rodent model to measure vaccine efficacy in a preclinical setting; hence, we advocate for its continued use to assess novel vaccine approaches.

The design of the multi-stage vaccine candidate, ProC6C, was guided by a series of functional mAbs against both sporozoite and transmission stages (15, 32–35). The findings reported here confirm and extend previous findings in rodent immunization studies that ProC6C elicit high levels of bi-functional antibodies with the capacity to inhibit sporozoite invasion of the liver and at the same time parasite multiplication in the infected mosquito. Reducing malaria transmission is considered essential for malaria elimination initiatives, largely because most malaria cases are asymptomatic and therefore relatively unaffected by current malaria control interventions that target reduction of the disease burden. Transmission blocking vaccine (TBV) candidates, including Pfs230-EPA and ProC6C (16, 36), are therefore high priority tools for malaria eradication (37). Successfully combining AIV (anti-infection vaccine) and TBV (transmission-blocking vaccine) as a multi-stage vaccine will transform the impact of vaccination from protection of vulnerable groups to a reduction of disease burden in the population by reducing the incidence of *P. falciparum* infection in both human and mosquito populations. The TBV component in a combination vaccine is particularly important as they may block selection for AIV escape mutants as well as reduce the spread of artemisinin resistant strains which are an emerging threat in malaria endemic countries.

Taken together, Phase 1 clinical trial results have demonstrated that ProC6C-AlOH/Matrix-M elicits transmission reducing antibodies (>80% TRA in >65% of volunteers), as well as functional PfCSP-specific antibodies. We have focused the analysis reported here on the ProC6C-AlOH/Matrix-M formulation as it elicited the highest level of transmission reducing activity and the highest anti-CSP titers, however we acknowledge that high titers do not always equate to protection as exemplified by the early development of RTS,S (38).

To our knowledge, ProC6C-AlOH/Matrix-M is the first subunit vaccine that elicited bi-functional antibodies against sporozoite and transmission stages in humans. Taken together with prior findings of ProC6C-AlOH/Matrix-M being safe and well tolerated in three African clinical trials, these new findings support continued clinical evaluation. Further testing of the standard three-dose (0,1,2) monthly regimen in a Controlled Human Malaria Infection (CHMI) study in an adult population in Mali (PACTR202404598604620) is in progress to ascertain whether ProC6C elicit protection against the human infection.

## Data Availability

The raw data supporting the conclusions of this article will be made available by the authors, without undue reservation.
