# Supplementary material for: ProC6C, a novel multi-stage malaria vaccine, elicits functional antibodies against the minor and central repeats of the Circumsporozoite Protein in human adults
Source: Front Immunol. 2024 Nov 1;15:1481829. doi: 10.3389/fimmu.2024.1481829 (PMC11563800; doi:10.3389/fimmu.2024.1481829)
Supplement: Supplementary file 1 [file DataSheet1.docx]

ProC6C, a novel multi-stage malaria vaccine, elicits functional antibodies against the minor and central repeats of the Circumsporozoite Protein in human adults

**Running Title:** ProC6C elicits CSP antibodies in humans

**Jordan Plieskatt^1†^, Ebenezer Addo Ofori^1,2†^, Mohammad Naghizadeh^1,2^, Kazutoyo Miura^3^, Yevel Flores-Garcia^4^, Nis Borbye-Lorenzen^1^, Alfred B. Tiono^5^, Kristin Skogstrand^1^, Issaka Sagara^6^, Fidel Zavala^4^, Michael Theisen^1,2,*^**

**Supplementary Figures**

**Supplementary Figure S1. Assay qualification of peptides.**  Anti-CSP mAbs **(A)** mAb317 and **(B)** mAbL9 loaded at various concentration (16, 8, 4, 2, 1, 0.5 µg/mL) were tested against major **(A)** and minor **(B)** peptides respectively to obtain antibody association (ka), dissociation (kd) and equilibrium (KD) rate constant values.

1. **(B)**

**
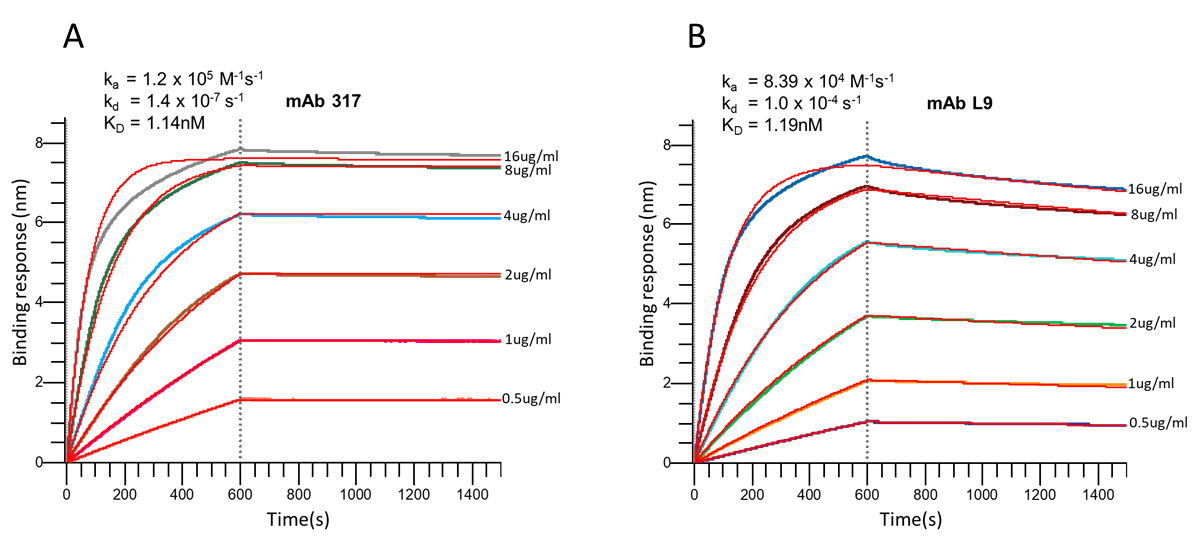
**

**Supplementary Figure S2**

Major and minor repeat antibodies elicited by Proc6C-AlOH. IgG was evaluated by peptide ELISA using major repeat peptide: (NANP)_6_ and minor repeat peptide: NANPNVDPNANPNVDP as plate antigens. **(A)** Anti-major repeat IgG levels (D0, D70, D180) and **(B)** Anti-minor repeat IgG levels (D0, D70, D180) from individuals receiving 3 vaccinations of ProC6C-AlOH on D0, D28, D56 are given as (**A)** 311 or **B)** L9 equivalences (µg/ml). The GMT for each day is shown by solid line. Statistical significance is indicated between days by one-way ANOVA with Turkey’s multiple comparison test. RTS, S/AS01 pools as in Figure 1 indicated by shades of orange circles. Anti-peptide IgG (Y axis) plotted against full-length CSP IgG (X axis) for D70 **(C)** and D180 **(D)**. Correlation coefficients (r) are shown for major (dark red) and minor (light red). Simple linear regressions are indicated by solid line.

# Supplementary Figure S3. BLI responses against major and minor repeats. The major and minor repeat responses by BLI assay for (A) Major and (B) Minor peptides of D0, D70, and D180 samples. Median indicated by line. RTS,S/AS01 pools loaded as described previously. Statistical significance was determined by One Way ANOVA with multiple comparisons test.

**Supplementary Figure S4**. BLI responses (Y-axis) relative to ELISA measured responses (X-axis) to the major (A-C) and minor (D-F) repeat peptides. Global analysis of both ProC6C-AlOH and ProC6C-AlOH/Matrix-M together on the same plots.
